# Supplementary material for: Conditional chemoconnectomics (cCCTomics) as a strategy for efficient and conditional targeting of chemical transmission
Source: eLife. 2024 Apr 30;12:RP91927. doi: 10.7554/eLife.91927 (PMC11060718; doi:10.7554/eLife.91927)
Supplement: Figure 3—source data 3. [file elife-91927-fig3-data3.zip › Figure 3 source data3 part 1.zip!/Figure 3-Source data 3-part1/1071-1-CRISPResso_Report_osfbjvga.zip!/Nucleotide_percentage_quilt.pdf]

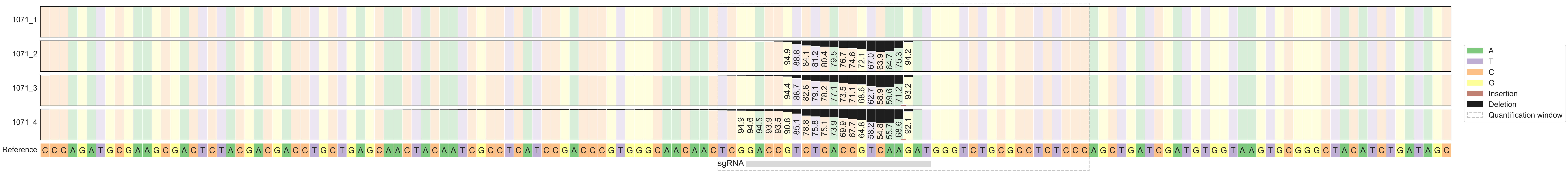

1071_1
1071_2
94.9 88.8 84.1 81.2 80.4 79.5 76.7 74.6 72.1 67.0 63.9 64.7 75.3 94.2
1071_3
94.4 88.7 82.6 79.1 78.2 77.1 73.5 71.1 68.6 62.7 58.9 59.6 71.2 93.2
1071_4
94.9 94.6 94.5 93.9 93.5 90.8 85.1 78.8 75.8 75.1 73.9 69.9 67.7 64.8 58.2 54.8 55.7 68.6 92.1
Reference
CCCAGATGCGAAGCGACTCTACGACGACCTGCTGAGCAACTACAATCGCCTCATCCGACCCGTGGGCAACAACTCGGACCGTCTCACCGTCAAGATGGGTCTGCGCCTCTCCCAGCTGATCGATGTGGTAAGTGCGGGCTACATCTGATAGC
sgRNA
A
T
C
G
Insertion
Deletion
Quantification window
